# Concurrent Administration of COVID-19 and Influenza Vaccines Enhances Spike-Specific Antibody Responses

**DOI:** 10.1093/ofid/ofae144

**Published:** 2024-03-13

**Authors:** Susanna E Barouch, Taras M Chicz, Ross Blanc, Domenic R Barbati, Lily J Parker, Xin Tong, Wenjun Li, Ryan P McNamara

**Affiliations:** Ragon Institute of MGH, MIT, and Harvard, Cambridge, Massachusetts, USA; Ragon Institute of MGH, MIT, and Harvard, Cambridge, Massachusetts, USA; Ragon Institute of MGH, MIT, and Harvard, Cambridge, Massachusetts, USA; Ragon Institute of MGH, MIT, and Harvard, Cambridge, Massachusetts, USA; Ragon Institute of MGH, MIT, and Harvard, Cambridge, Massachusetts, USA; Ragon Institute of MGH, MIT, and Harvard, Cambridge, Massachusetts, USA; Department of Public Health, Center for Health Statistics and Biostatistics, University of Massachusetts at Lowell. Lowell, Massachusetts, USA; Ragon Institute of MGH, MIT, and Harvard, Cambridge, Massachusetts, USA

**Keywords:** bivalent, COVID-19, influenza, vaccine, XBB.1.5

## Abstract

**Background:**

The bivalent COVID-19 mRNA boosters became available in fall 2022 and were recommended alongside the seasonal influenza vaccine. However, the immunogenicity of concurrent vs separate administration of these vaccines remains unclear.

**Methods:**

Here, we analyzed antibody responses in health care workers who received the bivalent COVID-19 booster and the influenza vaccine on the same day or on different days through systems serology. Antibody-binding and functional responses were characterized at peak responses and after 6 months following vaccination.

**Results:**

IgG1 and neutralization responses to SARS-CoV-2 XBB.1.5 were higher at peak and after 6 months following concurrent administration as compared with separate administration of the COVID-19 and influenza vaccines. While similar results were not observed for influenza responses, no interference was noted with concurrent administration.

**Conclusions:**

These data suggest that concurrent administration of these vaccines may yield higher and more durable SARS-CoV-2 neutralizing antibody responses while maintaining responses against influenza.

The bivalent COVID-19 mRNA vaccines encoded ancestral and BA.5 spike [1] , and subsequent Omicron lineages emerged that further escaped antibody recognition [2], including XBB strains [3, 4]. The rollout of the bivalent COVID-19 mRNA vaccines in fall 2022 coincided with the seasonal influenza vaccines. Previous work studying concurrent administration of seasonal influenza and ancestral COVID-19 vaccines, such as BNT162b2 and ChAdOx1, showed no interference in immune responses to either vaccine. Additionally, rates of adverse events were similar in this placebo-controlled study [5]. However, it has remained unclear how concurrent administration of the updated COVID-19 mRNA and influenza vaccines may affect the antibody profiles generated. Additionally, how the antibody profiles are sustained beyond peak immunogenicity [6–8] is unclear when the 2 vaccines are coadministered.

Here we profiled antibody responses of health care workers who received the bivalent COVID-19 mRNA booster and the seasonal influenza vaccine on the same day or different days. We analyzed responses to the predominant variant at the time of the study: XBB.1.5 spike. We observed significantly higher IgG1 responses and neutralization to XBB.1.5 at peak and after 6 months. While IgG1 responses to influenza antigens did not display a phenotype as XBB.1.5 spike, no immune interference was noted when the influenza vaccine was concurrently administered with the bivalent COVID-19 booster. Our study suggests an immunologic benefit to concurrent vaccination with these 2 vaccines for spike-specific antibody responses.

## METHODS

### Experimental Outline and Study Participants

Participants were enrolled as a part of the Massachusetts Consortium on Pathogen Readiness with informed consent. Individuals were divided into participants who received an influenza vaccine on the same day as the bivalent COVID-19 mRNA vaccine or those who received the 2 vaccines on different days within 4 weeks. Vaccines were administered September to December 2022. Serum samples were obtained 3 to 4 weeks and 6 months after the COVID-19 booster. The median ages were 36 years (range, 26–62) for those who received the vaccines concurrently and 39 years (range, 23–72) for those who received the vaccines on different days. Groups were predominantly female (86% and 80%, respectively) and had similar baseline medical conditions. Of the group that had a bivalent mRNA boost, 15 were administered Pfizer-BioNTech and 29 Moderna. Individuals who received the influenza vaccine before the COVID-19 booster acquired it a median 8.4 days before (range, 1–28), and those who received the influenza vaccine after the COVID-19 booster got it a median 13.2 days after (range, 2–29).

The flu vaccines administered during this study period were Fluarix and Fluzone. The antigenic composition of the 2022–2023 influenza vaccine was used to perform antigen-binding profiling, along with other influenza antigens [9, 10]. Neither of these vaccines contains a characterized adjuvant [11].

### Antibody-Binding Profiling

Antibody subclasses, isotypes, and Fc receptor–binding antibodies were assayed for binding to antigens listed in Supplementary Table 1 and described elsewhere [12]. Assays for SARS-CoV-2 spike and influenza antigens were done separately. The primary immunologic end point for SARS-CoV-2 responses was antibody responses to the predominant circulating SARS-CoV-2 variant at the time of this study: XBB.1.5. Exploratory end points were antibody responses to other SARS-CoV-2 variants. The breadth of antibody subclass and isotype binding was quantified by standardizing each subclass and isotype to Wu-1 spike binding for administration of the vaccinations on different days (Supplementary Figure 1). Antibody-binding responses to influenza antigens were to the hemagglutinin (HA) components of the quadrivalent vaccine administered during the 2022–2023 season. Other influenza antigens and components of previous seasonal vaccinations were also used for exploratory analyses.

### Antibody Functionality Characterization

Pseudovirus neutralization with serum from the cohort was performed as previously described [3]. Antibody effector–mediated functions, such as antibody-dependent cellular phagocytosis by monocytes and neutrophil phagocytosis, were done as previously described [6]. For antibody-dependent cellular and neutrophil phagocytosis, results were quantified with a previously validated flow cytometry–based assay, and readouts were quantified as a “phagoscore” (see McNamara et al [12]).

### Quantification and Statistical Analysis

All figures and statistics were done in R Studio version 6.0 or Prism version 10 (GraphPad). For correlation plots, a Spearman rank correlation was calculated against individual pairings and plotted as a heat map. For comparisons of responses, an initial Wilcoxon rank sum test was performed, followed by a Bonferroni correction for multiple comparisons when appropriate [13]. For SARS-CoV-2 responses, the primary end point was the antibody response to the predominant circulating SARS-CoV-2 variant at the time of the study: XBB.1.5 [3, 14, 15]. Antibody responses to the 2 components of the bivalent mRNA vaccine booster, Wu-1 and BA.5 spike [16], were assessed as an exploratory end point. For influenza responses, antigens belonging to components of the seasonal influenza vaccine—influenza A and B—were assessed.

## RESULTS

### Concurrent Bivalent COVID-19 and Influenza Vaccination Led to Higher XBB.1.5 Spike IgG1 Responses

**Figure 1.**
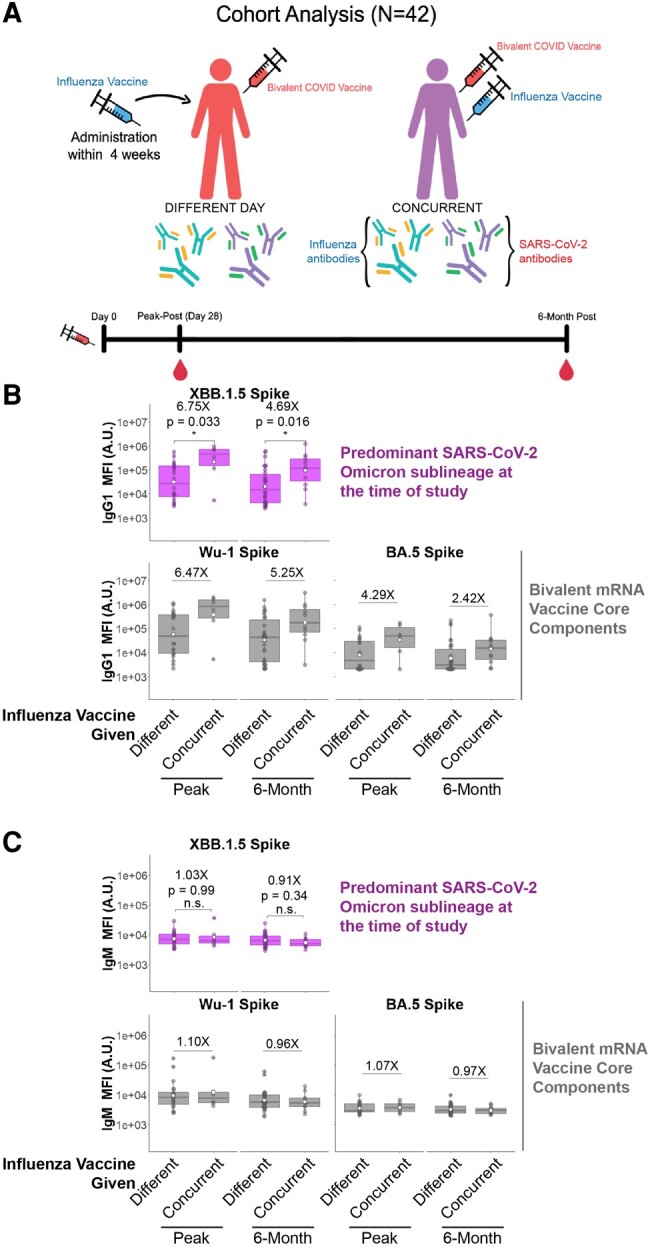
Concurrent bivalent COVID-19 mRNA and influenza boosters induce more durable IgG1 responses to spike. *A*, Cohort analyzed in this study. Participants were divided into those who received a bivalent mRNA COVID-19 booster and flu vaccine on the same day (concurrently) or different days. Blood was drawn at peak immunogenicity (2–4 weeks) and 6 months after the bivalent COVID-19 mRNA booster. *B*, IgG1 antibody responses to the predominantly circulating COVID-19 spike variant at the time of this study XBB.1.5 (top) as well as the ancestral (Wu-1) and Omicron BA.5 (bottom) spikes that represent the vaccine immunogens. *C*, IgM antibody responses were quantified similarly to panel *B* and serve as a control to assess de novo antibody affinity maturation. White circle, mean; line, median; box, IQR; error bars, 95% CI. Fold differences and *P* values are shown. *P < .05. ns, not significant. Mann-Whitney *U* test/Wilcoxon rank sum test.

A cohort of 42 healthcare workers was followed longitudinally after bivalent COVID-19 mRNA boosting in fall 2022. Sera were evaluated at weeks 3 to 4 after boosting (peak immunogenicity) and at month 6 after boosting. The cohort was divided into individuals who received the COVID-19 booster and the influenza vaccine on the same day (n = 12) or on different days (n = 30; Figure 1*A*). The primary objective was to assess antibody responses to the predominantly circulating SARS-CoV-2 variant at the time of the study XBB.1.5.

IgG1 responses to XBB.1.5 spike in individuals who received the bivalent mRNA COVID-19 vaccine concurrently with the influenza vaccine were 6.75-fold higher at peak and 4.69-fold higher at 6 months as compared with those who were administered the 2 vaccines on different days (Figure 1*B*, purple box and whiskers; *P* = .033 and *P* = .016 at peak and 6 months, respectively). Antibody responses to Wu-1 and BA.5 spike followed similar trends when the bivalent mRNA COVID-19 vaccine was administered concurrently with the influenza vaccine (6.47X and 4.29X for Wu-1 and BA.5 spike at peak post and 5.25X and 2.42X for Wu-1 and BA.5 spike at 6 months; Figure 1*B*, gray bars). In individuals who received the vaccines on different days, no differences were observed according to vaccination order (Supplementary Figure 2A and 2B). No IgG1 responses were observed to Ebola virus glycoprotein (negative control; Supplementary Figure 2C).

**Figure 2.**
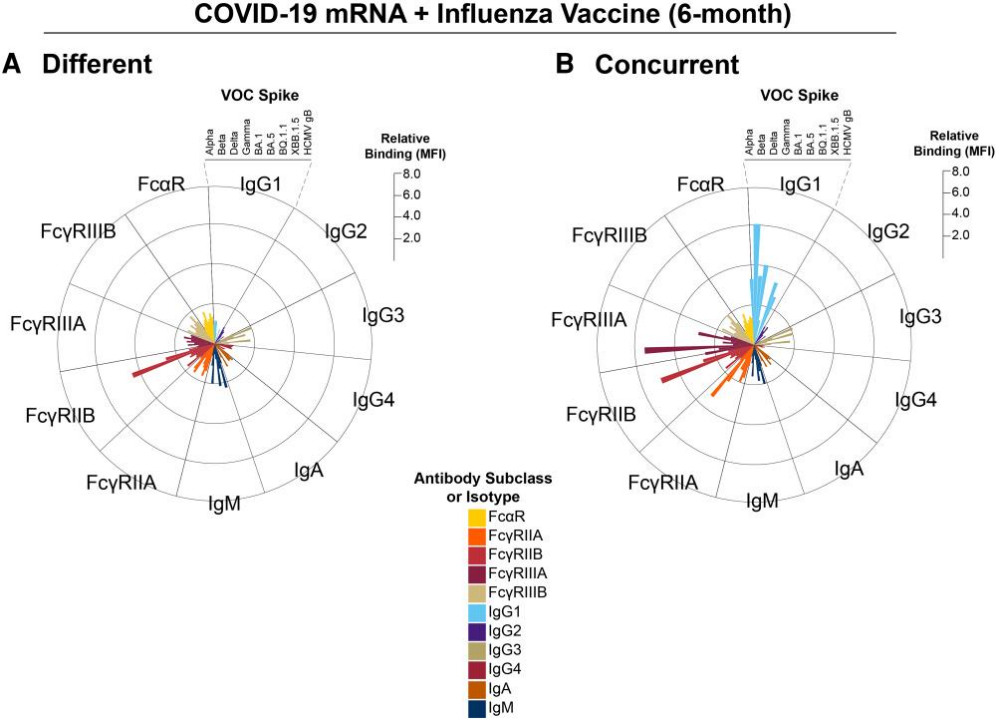
Concurrent COVID-19 mRNA and influenza vaccination selectively expands IgG1-binding breadth to multiple spike variants. *A*, Radar plot shows relative binding of individual antibody isotypes, subclasses, and FcγR- and FcαR-binding antibodies to identified SARS-CoV-2 and control antigens. Individual bars represent the median fluorescence activity (MFI) of a specific feature standardized to that antibody subclass/isotype response to Wu-1 spike for individuals who received the bivalent mRNA and influenza vaccine on different days 6 months after the bivalent COVID-19 booster. The scale on the right represents fold MFI increase relative to Wu-1 spike for each antibody feature. *B*, Radar plot shows relative binding of individual antibody isotypes, subclasses, and FcγR- and FcαR-binding antibodies to the identified SARS-CoV-2 and control antigens for individuals who received the bivalent COVID-19 and influenza vaccine concurrently. Individual bars represent the MFI of a specific feature standardized to that antibody/isotype response to Wu-1 spike for individuals who received the bivalent mRNA and influenza vaccine on different days 6 months after the bivalent COVID-19 booster. The Wu-1 spike responses of individuals who received the vaccines on different days were used as a standard to compare across groups. The scale on the right represents MFI increase. All antibody isotypes, subclasses, and FcγR- and FcαR-binding antibodies are shown in distinct colors, and a legend is shown at the bottom. FcαR, Fc-α receptor; FcγR, Fc-γ receptor; VOC, variant of concern.

No differences were observed in IgM responses (Figure 1*C*), suggesting that concurrent COVID-19 and influenza vaccination drove enhanced recall responses rather than de novo responses [12]. Other IgG subclasses were also quantified for binding to these antigens, including IgG2, IgG3, IgG4, and IgA. While trends were noticed, these comparisons were not statistically significant (Supplementary Figure 3).

### Concurrent Bivalent COVID-19 and Influenza Vaccinations Increased Spike-Specific IgG1 Breadth to Multiple Variants 6 Months Postvaccination

We next performed an exploratory analysis to assess if concurrent vaccination increased IgG1 binding breadth to SARS-CoV-2 spikes to multiple variants. These included Alpha, Beta, Delta, Gamma, and BQ.1.1, in addition to the previously tested spikes. Comparisons between concurrent and different vaccination days showed consistently increased IgG1 responses and sustained FcγRIIIA responses at 6 months to all these spike variants in individuals who received the vaccines concurrently (Figure 2). We also observed a more robust correlation between IgG1 and IgG3 with FcγRs at peak immunogenicity and 6 months in individuals who were administered the vaccines concurrently as compared with those who had the vaccines on different days (Supplementary Figure 4).

Antibody functions including pseudovirus neutralization and effector functions were then quantified. At peak and 6 months following vaccination, pseudovirus neutralization to BA.5 and XBB.1.5 was higher in those who received the 2 vaccines concurrently (Supplementary Figure 5A and 5B). This increase was not observed for effector functions, such as antibody-dependent cellular phagocytosis by monocytes and neutrophil phagocytosis to the bivalent vaccine components or to XBB.1.5 spike (Supplementary Figure 5C and 5D). Interestingly, subclass quantitation showed a consistently lower IgG4 as a fraction of the whole IgG repertoire for individuals who had the 2 vaccines concurrently (Supplementary Figure 6).

No significant differences were observed for antibody responses to SARS-CoV-2 nucleocapsid, arguing against differential infection affecting humoral profiles (Supplementary Figure 7).

### Concurrent Bivalent COVID-19 and Influenza Boosting Did Not Affect Influenza HA Antibody Responses

As we defined peak responses in relation to when participants received the bivalent mRNA COVID-19 vaccine, we did not capture peak responses to the influenza vaccine in this study. However, we assayed antibody responses to influenza HA at approximately 6 months following immunization.

Unlike SARS-CoV-2 spike responses, we did not detect differences in influenza HA responses as a consequence of vaccination on the same day or different days (Supplementary Figure 8). We tested for components of the 2022–2023 seasonal influenza vaccine, including Influenza A Darwin 9, Influenza A Victoria 2570, Influenza A Wisconsin 588 pdm09, Influenza B Austria 1359, and Influenza B Phuket 3073 (for the quadrivalent vaccine) [9].

## DISCUSSION

This study shows that concurrent administration of the bivalent COVID-19 booster and the inactivated influenza vaccine on the same day resulted in higher XBB.1.5 spike-specific binding IgG1 responses at peak and 6 months as compared with administration of these vaccines on separate days. Moreover, neutralizing antibodies toward BA.5 and XBB.1.5 were higher at the peak and 6-month time points when the bivalent booster was administered concurrently as compared with different days.

Safety profiles of concurrent COVID-19 and influenza vaccination have been reported [17], but limited data exist on the durability of antibody responses following different vaccination schedules. Lazarus et al found no interference in antibody generation to either influenza HA or SARS-CoV-2 spike when the ancestral COVID-19 vaccines were coadministered with seasonal influenza vaccines [5]. Another previous report analyzing quadrivalent influenza and mRNA-1273 vaccines showed no antigen interference or safety concerns [18]. Our data extend these prior studies by evaluating the bivalent mRNA COVID-19 vaccine with the seasonal influenza vaccine in the context of widespread population immunity in 2022–2023. It has been estimated that over half the US population was infected during the BA.1 wave [19]. Moreover, we show that the enhanced antibody responses observed at peak were also durable for at least 6 months.

IgG1 is the most abundant serum IgG subclass and is capable of neutralizing and nonneutralizing functions. A correlate of protection against COVID-19 of neutralizing antibodies has been reported, but this was studied only for the ancestral Wu-1 virus [20]. Other reports have suggested that Fc effector functions may be required for protection against Omicron variant spike [12, 21, 22]. While neutralization titers to XBB.1.5 in our study were higher in those who received the COVID-19 and influenza vaccines concurrently, we did not find a similar increase in effector-mediated functions. This IgG1 and neutralization response appeared to be a recall response [12] as no differences in IgM were noted.

Previous reports showed that mRNA COVID-19 vaccine boosting can disproportionately expand IgG4 responses [23, 24]. We did not find evidence that concurrent administration of the mRNA COVID-19 boosters and seasonal influenza vaccines affected IgG4 expansion. This is in agreement with previous literature that showed a lack of interference when these 2 vaccines were coadministered [5].

In summary, our results suggest potential benefits of concurrent administration of the COVID-19 mRNA vaccines and the seasonal influenza vaccine for induction and durability of spike-specific IgG1 and neutralizing antibody responses. Because of the expected seasonality of SARS-CoV-2 and influenza, both vaccines will likely continue to be recommended. Our results suggest that concurrent administration of these vaccines should be considered a strategy to potentiate antibody responses to the COVID-19 vaccine and possibly improve vaccine effectiveness [20, 25].

### Limitations

A limitation of our study is the small size of this cohort, which primarily consisted of health care workers and may not reflect the general population. Larger future studies are therefore needed. Future studies should also involve a broader age range than our cohort, including children and elderly adults [26–29]. Another limitation was that the peak responses were defined relative to mRNA COVID-19 vaccination and we did not capture peak influenza responses. Moreover, how adenovirus- and protein-based COVID-19 vaccine boosters [30–32] affect influenza responses generated by inactivated and live-attenuated vaccines [10] remains to be determined. Last, we were unable to assess durability beyond 6 months, although this time frame covers a typical influenza season.

## Supplementary Data

Supplementary materials are available at *Open Forum Infectious Diseases* online. Consisting of data provided by the authors to benefit the reader, the posted materials are not copyedited and are the sole responsibility of the authors, so questions or comments should be addressed to the corresponding author.

## Supplementary Material

ofae144_Supplementary_Data

## Notes

***Acknowledgments.*** We thank Nancy Zimmerman, Mark and Lisa Schwartz, an anonymous donor (general financial contribution to the Ragon Institute), and Terry and Susan Ragon for their donations to the Ragon Institute.

***Author contributions.*** Conceptualization: S. E. B., X. T., and R. P. M. Data curation: S. E. B., T. M. C., D. R. B., R. B., L. J. P., and R. P. M. Formal analysis: S. E. B., T. M. C., D. R. B., R. B., L. J. P., W. L., and R. P. M. Funding acquisition: R. P. M. Investigation: S. E. B., T. M. C., D. R. B., R. B., L. J. P., X. T., and R. P. M. Methodology: S. E. B., T. M. C., D. R. B., R. B., L. J. P., X. T., and R. P. M. Project administration: X. T. and R. P. M. Resources: R. P. M. Software: S. E. B., L. J. P., and R. P. M. Supervision: X. T. and R. P. M. Validation: S. E. B., T. M. C., D. R. B., R. B., L. J. P., W. L., and R. P. M. Visualization: S. E. B. and R. P. M. Writing–original draft: S. E. B., T. M. C., D. R. B., R. B., and R. P. M.

***Disclaimer.*** The conclusions of the results presented here are those of the authors and do not purport to represent those of the funders and funding agencies.

***Patient consent statement.*** All participants were enrolled as a part of the Massachusetts Consortium on Pathogen Readiness biorepository cohort with approval from the institutional review board. All participants provided informed consent.

***Financial support*.** This work was supported by the Massachusetts Consortium of Pathogen Readiness, National Institutes of Health (1P01AI165072–01, 1P01AI168347–01, 4U01CA260476–02, CIVIC 75N93019C00052), and Bill and Melinda Gates Foundation (INV-031624 and INV-001650) to the Systems Serology Laboratory of R. P. M. Work at the Ragon Institute is also supported by M. and L. Schwartz and T. and S. Ragon,
