# Supplementary material for: Concurrent Administration of COVID-19 and Influenza Vaccines Enhances Spike-Specific Antibody Responses
Source: Open Forum Infect Dis. 2024 Mar 13;11(4):ofae144. doi: 10.1093/ofid/ofae144 (PMC10986856; doi:10.1093/ofid/ofae144)
Supplement: ofae144_Supplementary_Data [file ofae144_supplementary_data.docx]

**Supplementary Information**

Supplementary Information includes Figures 1-8 and their corresponding captions, and Supplementary Table 1.

**Supplementary Figure 1**


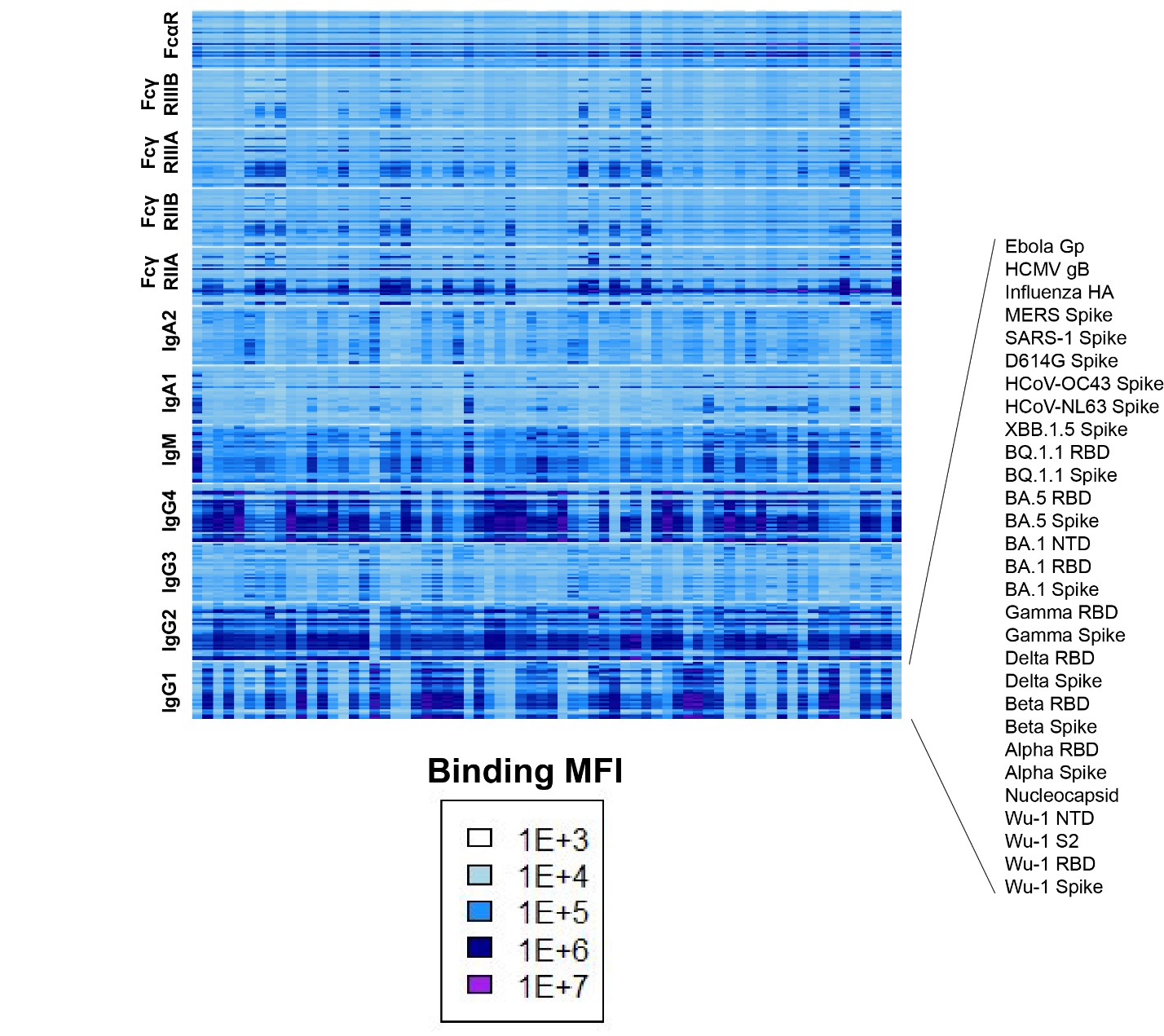


**Supplementary Figure 1. Heatmap of antibody binding to antigens.**

Binding profiling was done for antibody isotypes, subclasses, and FcRs shown on the left. Each column represents a single anonymized individual. Each row represents the antibody’s binding to the antigen (order list shown on the right). Binding was quantified through median fluorescence intensity (MFI) as arbitrary units, and a scale is shown at the bottom.

**Supplementary Figure 2**

**
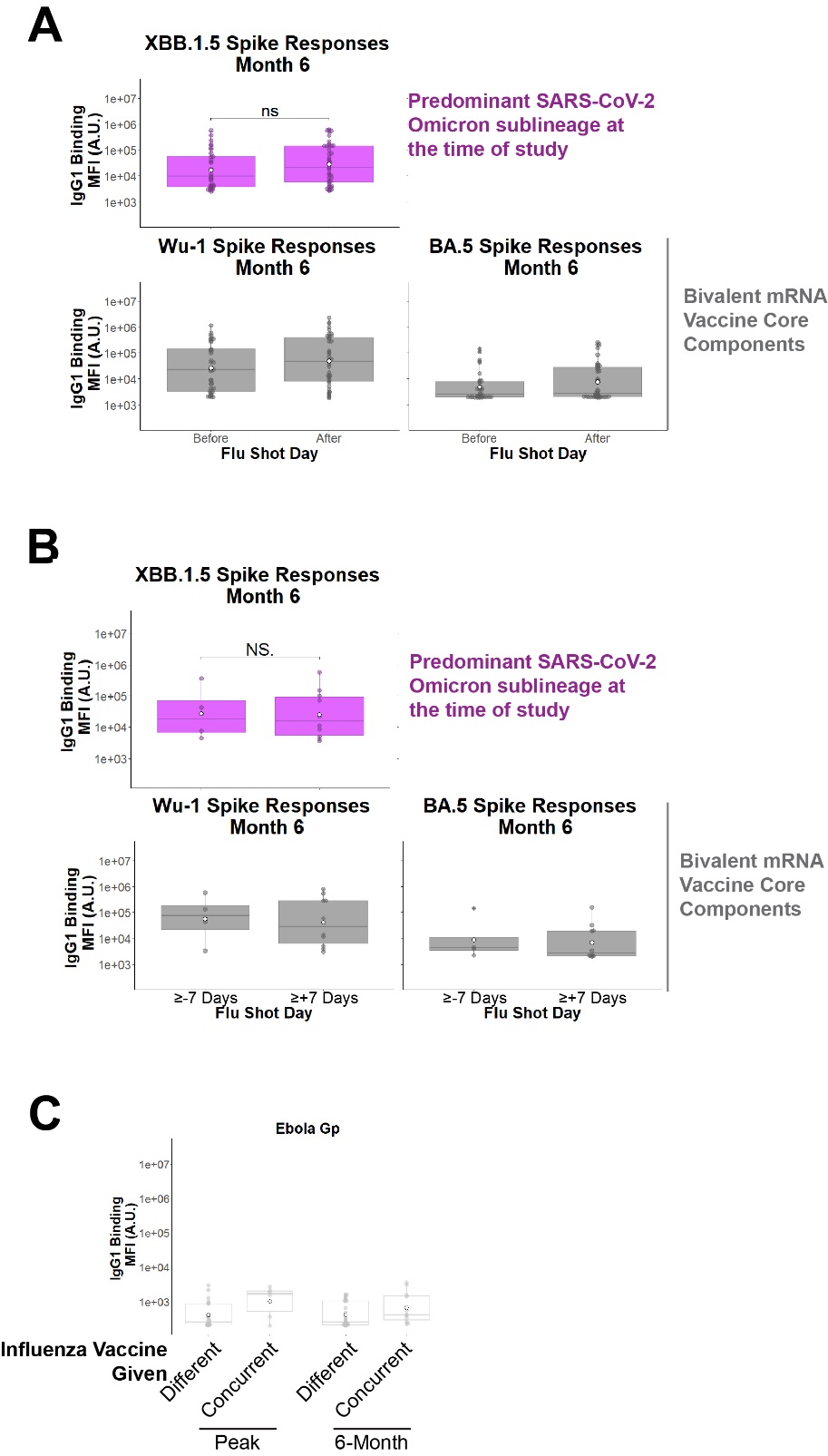
**

**Supplementary Figure 2. Receiving the influenza vaccine before or after the COVID-19 bivalent booster does not change 6 month responses.**

A. IgG1 binding to XBB.1.5 Spike and the two components of the bivalent COVID-19 booster, ancestral (Wu-1) Spike and Omicron BA.5 Spike were quantified at 6 months post-vaccination based on receiving the influenza vaccine before or after the bivalent COVID-19 booster.

B. Same as A, but only selecting for individuals that received the influenza vaccine ± >7 days from the bivalent COVID-19 booster.

C. Non-specific IgG1 binding is unaffected by concurrent vs. different day administration of the COVID-19 bivalent booster and influenza vaccine as determined by binding to the Ebolavirus glycoprotein (Gp).

For all comparisons, * = p<0.05, ns = not statistically significant, **Mann–Whitney U test / Wilcoxon rank-sum test**.

**Supplementary Figure 3**


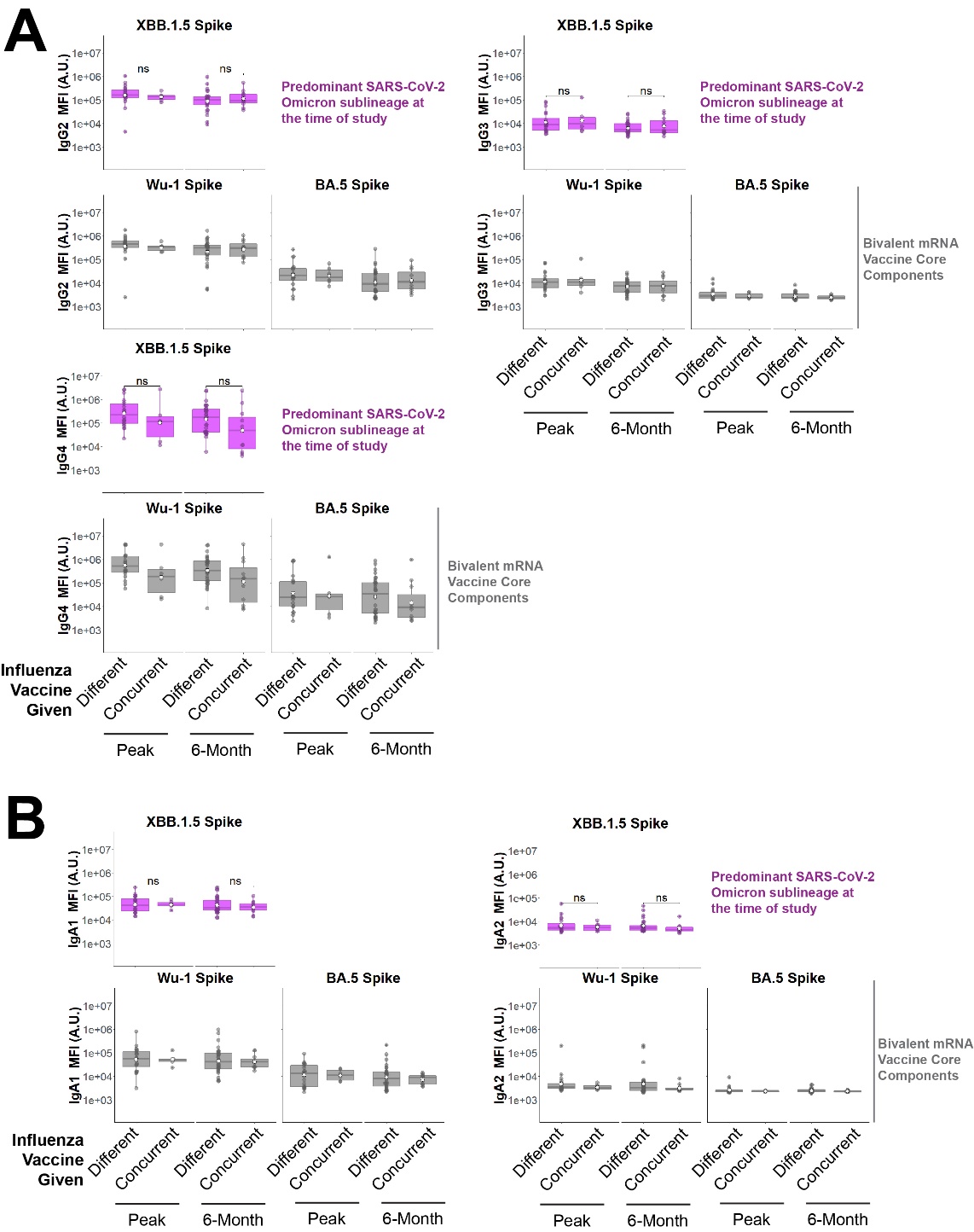


**Supplementary Figure 3. Binding antibodies other than IgG1 display no difference in binding capabilities from different or concurrent COVID-19 and influenza vaccinations.**

A. IgG2, IgG3, and IgG4 binding to XBB.1.5 Spike and the two components of the bivalent COVID-19 booster, ancestral (Wu-1), Omicron BA.5, were quantified for the indicated groups at peak immunogenicity and 6 months post-vaccination.

B. Same as A, but for IgA1 and IgA2.

For all comparisons, * = p<0.05, ns = not statistically significant, **Mann–Whitney U test / Wilcoxon rank-sum test**.

**Supplementary Figure 4**


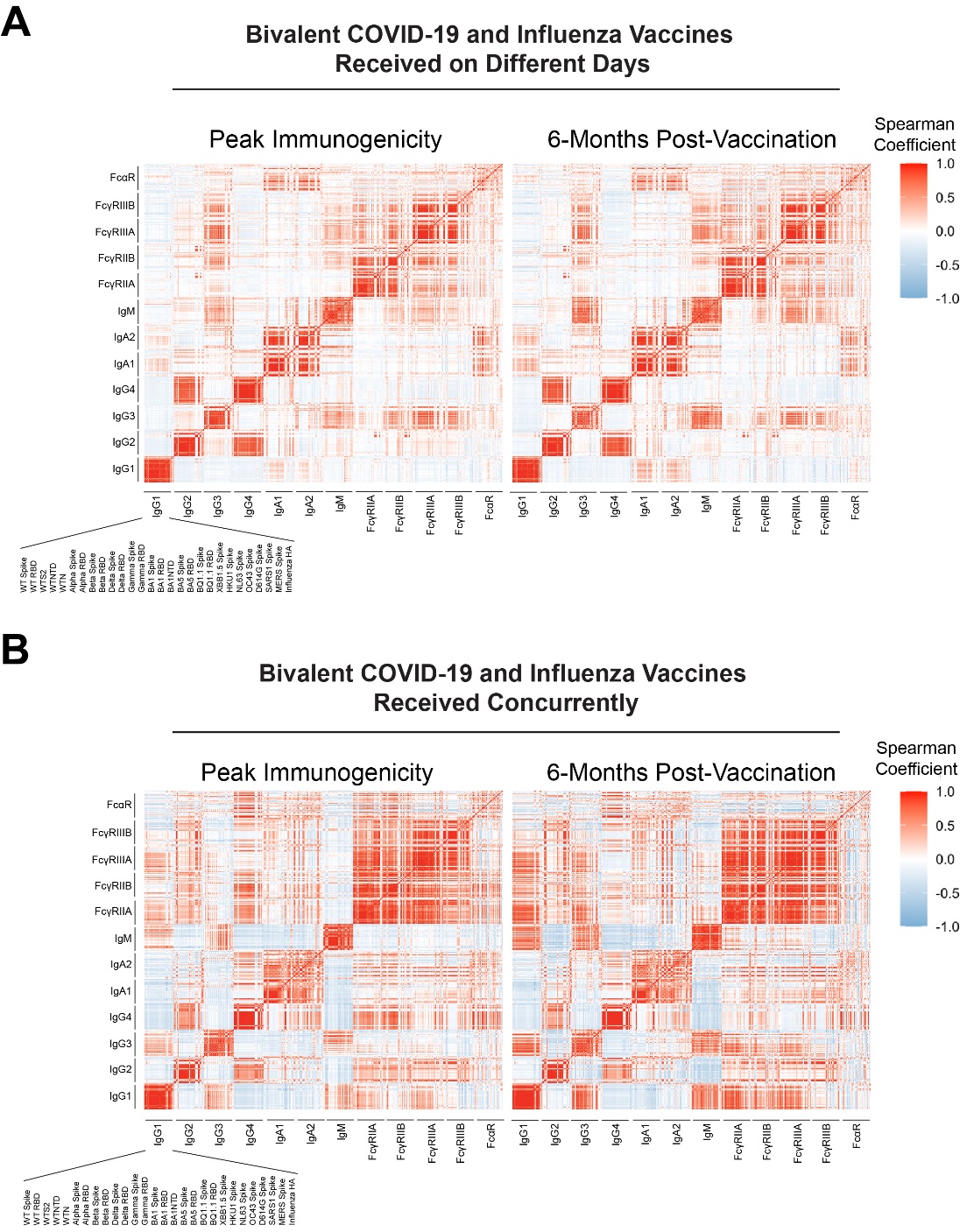


**Supplementary Figure 4. Correlation heatmaps of individuals show higher coordination between Fab-binding and FcR-binding antibodies for individuals who received the bivalent COVID-19 and influenza vaccines concurrently.**

A. Spearman’s correlation heatmap of individual antibody binding features with the indicated antigens at peak immunogenicity (left) and 6 months post-vaccination (right) for individuals that received the bivalent COVID-19 and seasonal influenza boosters on different days. Shown on the right is the heatmap legend for the correlation index for each pairwise comparison.

B. Same as (A), but for individuals that received the bivalent COVID-19 and seasonal influenza vaccine concurrently. The Heatmap legend is shown on the right.

**Supplementary Figure 5**

**
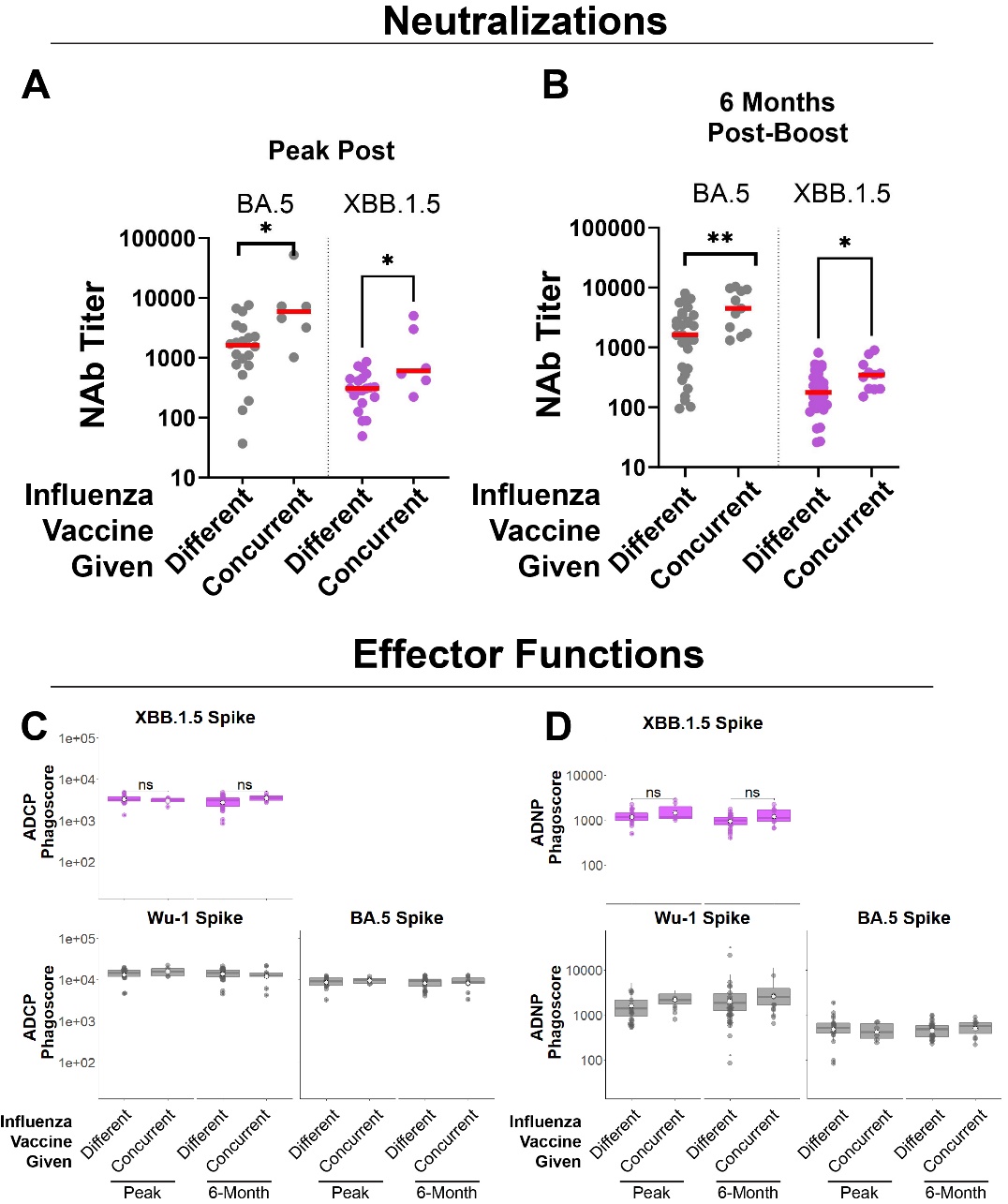
**

**Supplementary Figure 5. SARS-CoV-2 antibody functions in individuals who received the bivalent COVID-19 vaccine concurrently with an influenza vaccine or on different days.**

A. Pseudovirus neutralizing antibody (NAb) titers were quantified against (left) BA.5 Spike or (right) XBB.1.5 Spike. Sera was quantified for NAb at peak-post responses after bivalent COVID-19 booster for those who received the booster concurrently with an influenza vaccine or on different days. * = p < 0.05, ** = p < 0.01. Mann-Whitney U test.

B. Same as A, but for the 6 month post-boost timepoint for BA.5 and XBB.1.5 pseudovirus neutralization. * = p < 0.05, ** = p < 0.01. Mann-Whitney U test.

C. Antibody-dependent cellular phagocytosis by monocytes (ADCP), as quantified by a Phagoscore, was measured to XBB.1.5 Spike and the two components of the bivalent COVID-19 booster, Wu-1 and BA.5 Spike. ns = not statistically significant * = p < 0.05, ** = p < 0.01. Mann-Whitney U test.

D. Antibody-dependent neutrophil phagocytosis (ADNP), as quantified by a Phagoscore, was measured to XBB.1.5 Spike and the two components of the bivalent COVID-19 booster, Wu-1 and BA.5 Spike. ns = not statistically significant * = p < 0.05, ** = p < 0.01. Mann-Whitney U test.

**
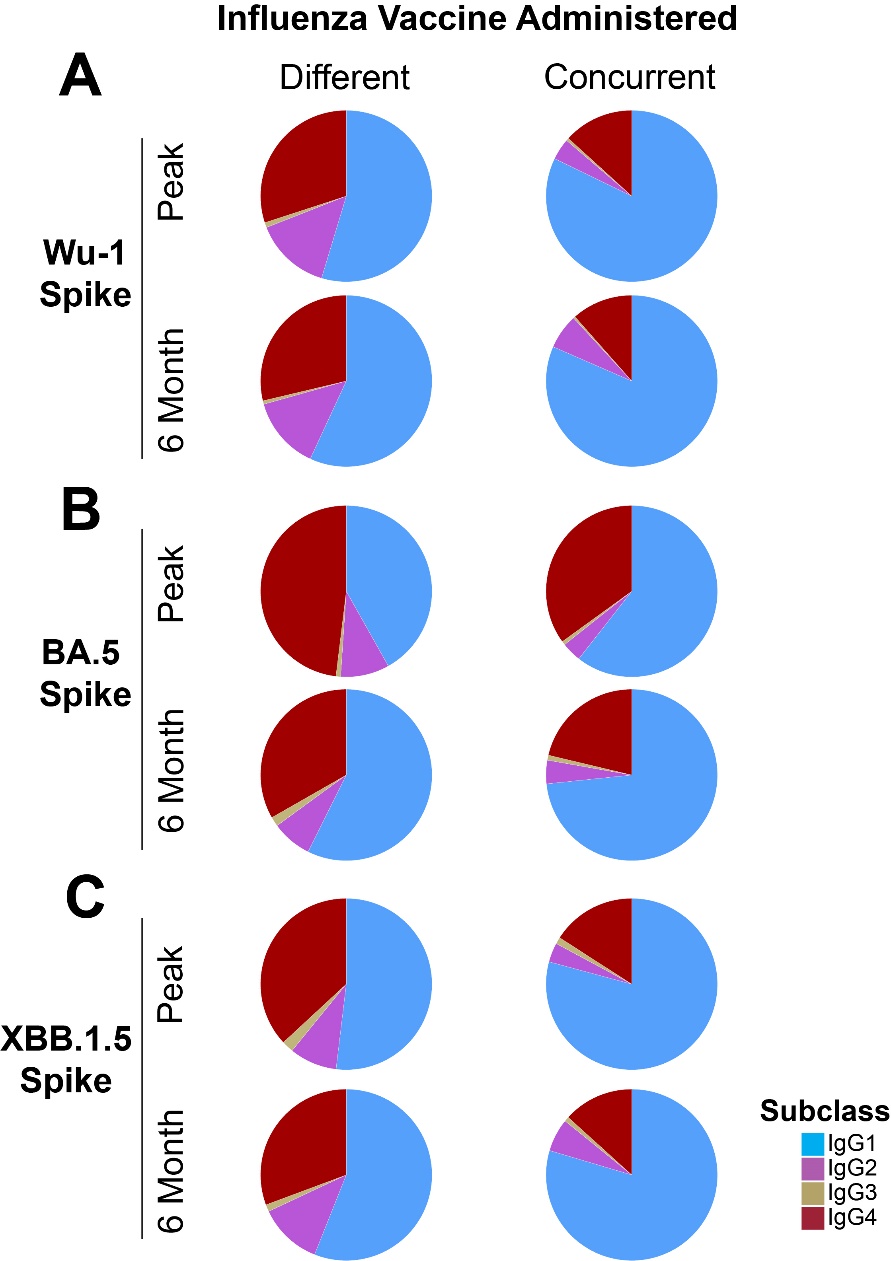
Supplementary Figure 6**

**Supplementary Figure 6. IgG subclass representation in individuals who received the bivalent COVID-19 booster concurrently with an influenza vaccine or on different days.**

A. Relative IgG subclass representation to Wu-1 Spike at Peak and 6-month post-boost responses were quantified in individuals who received the vaccines on different days or concurrently. Relative representation was quantified by taking the sum MFI of all subclasses, accounting for serum dilutions, as an area under the curve (AUC). Specific subclasses were then quantified for their contribution to the AUC and plotted as a pie chart. Color legend is shown in the bottom right.

B. Same as A, but for BA.5 Spike IgG responses

C. Same as A, but for XBB.1.5 Spike IgG responses.

**Supplementary Figure 7**

**
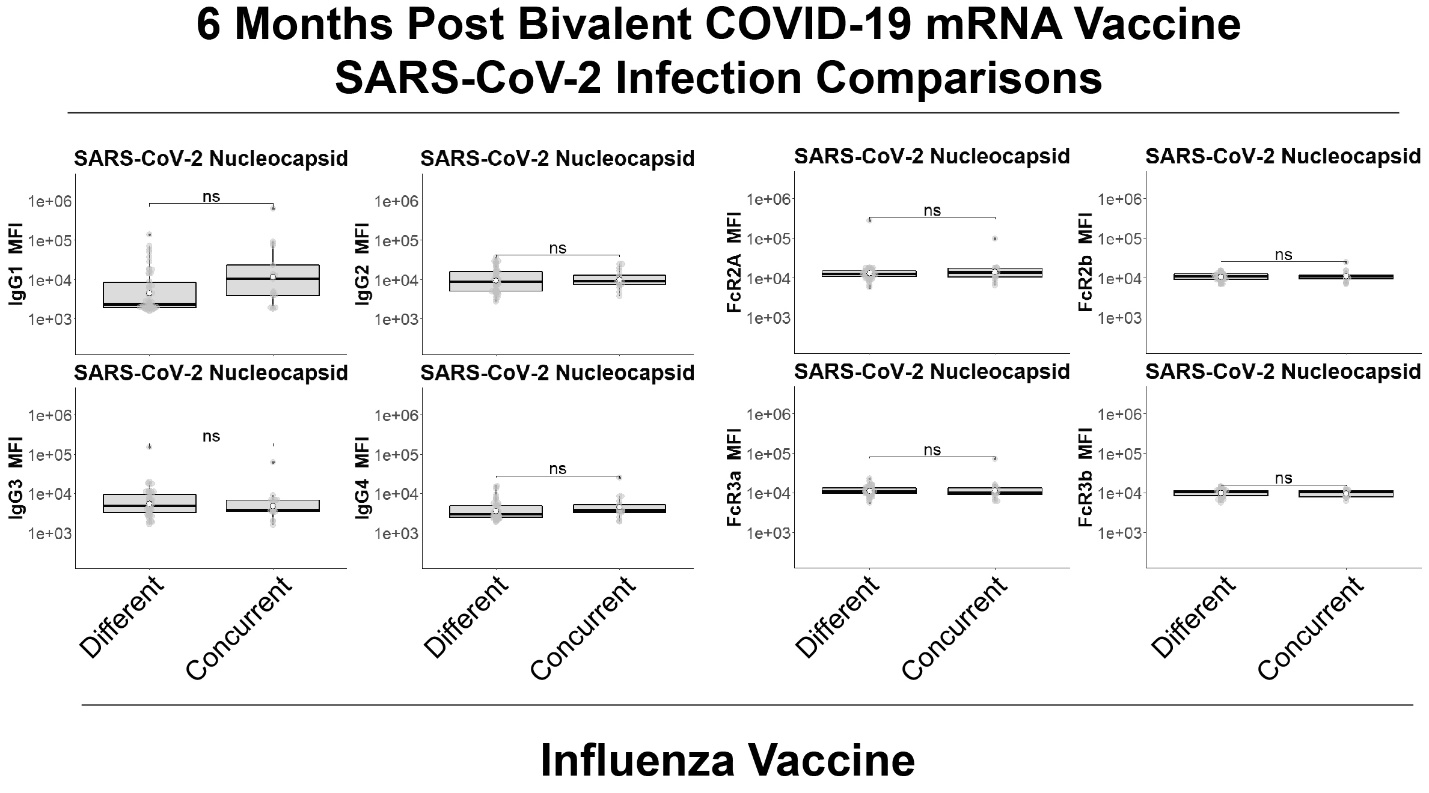
**

**Supplementary Figure 7. Infections during the observation period are not driving antibody profile distinctions between groups.**

IgG1-IgG4 and FcγRIIA – FcγRIIIB binding to Nucleocapsid was quantified for individuals who received the bivalent COVID-19 and influenza vaccines on different days or concurrently. Nucleocapsid was used as a bait antigen as it is not a component of the mRNA vaccine. Shown are responses at the 6 month time window to capture any indications of infection throughout the study period.

For all comparisons, * = p<0.05, ns = not statistically significant, **Mann–Whitney U test / Wilcoxon rank-sum test.**

**Supplementary Figure 8**

**
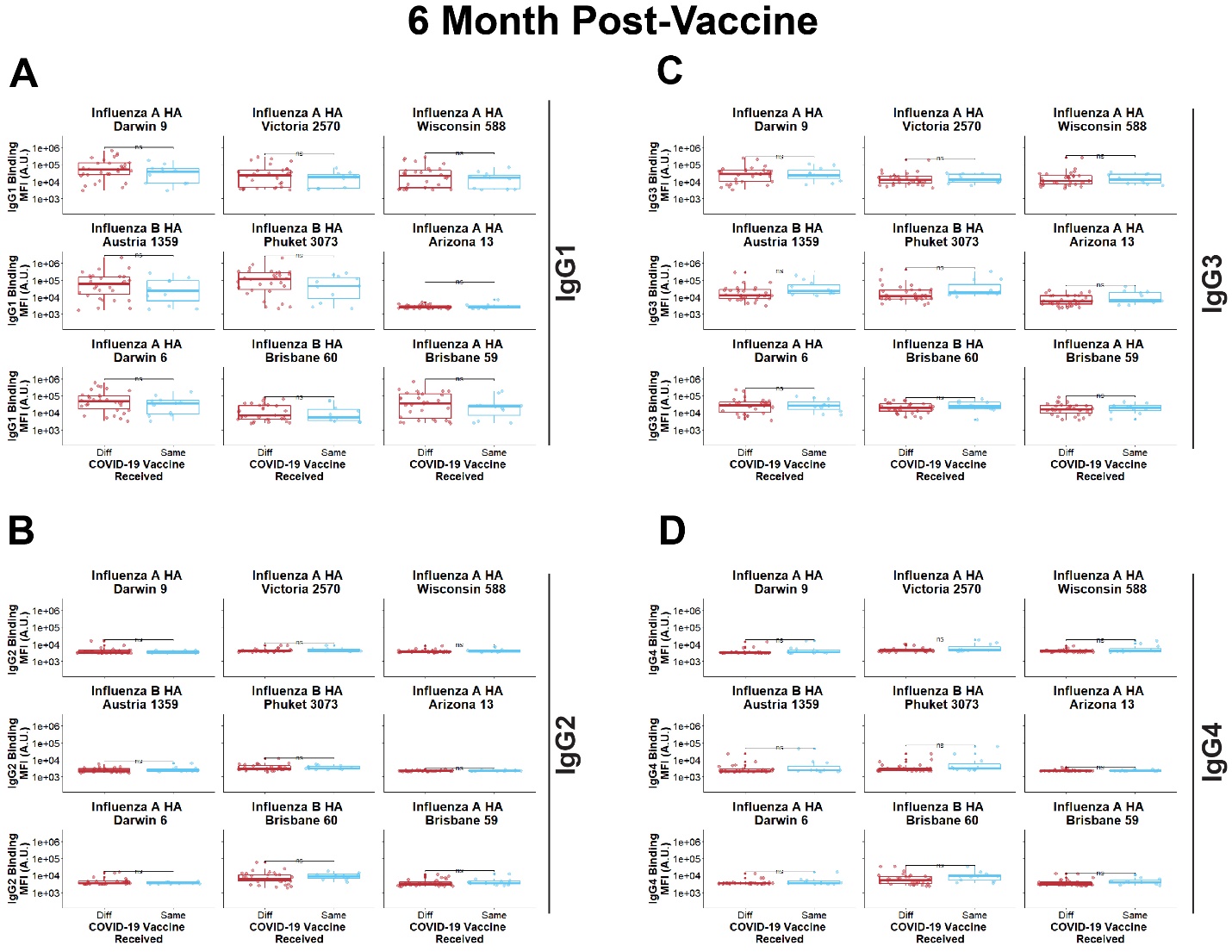
**

**Supplementary Figure 8. Concurrent COVID-19 and influenza boosting does not enhance or suppress 6 month influenza responses.**

A. IgG1 responses to an array of influenza antigens including those that were included in the 2022-2023 seasonal influenza vaccine were quantified in individuals who received the COVID-19 bivalent booster on different days or the same day (concurrently). For all comparisons, * = p<0.05, ns = not statistically significant, **Mann–Whitney U test / Wilcoxon rank-sum test** followed by a Bonferroni adjustment for multiple comparisons.

B. Same as A, but for IgG2.

C. Same as A, but for IgG3.

D. Same as A, but for IgG4

**Supplementary Table 1**

| REAGENT or RESOURCE | SOURCE | IDENTIFIER |
| --- | --- | --- |
| **Antibodies** | | |
| Mouse anti-human IgG1-PE | SouthernBiotech | 9054-09 |
| Mouse anti-human IgG2 PE | SouthernBiotech | 9070-09 |
| Mouse anti-human IgG3-PE | SouthernBiotech | 9210-09 |
| Mouse anti-human IgG4-PE | SouthernBiotech | 9200-09 |
| Mouse anti-human IgA1 | SouthernBiotech | 9130-09 |
| Mouse anti-human IgA2 | SouthernBiotech | 9140-09 |
| Mouse anti-human IgM | SouthernBiotech | 9020-09 |
| **Chemicals, peptides, and recombinant proteins** | | |
| **SARS-CoV-2 WT Spike** | Sino Biological | 40589-V08H4 |
| **SARS-CoV-2 WT S1** | Sino Biological | 40591-V08H |
| **SARS-CoV-2 WT RBD** | Sino Biological | 40592-V08H |
| **SARS-CoV-2 WT S2** | Sino Biological | 40590-V08B |
| **SARS-CoV-2 WT NTD** | Sino Biological | 40591-V49H |
| **SARS-CoV-2 D614G Spike** | Sino Biological | 40589-V08B4 |
| **SARS-CoV-2 Alpha Spike** | Sino Biological | 40589-V08H12 |
| **SARS-CoV-2 Alpha RBD** | Sino Biological | 40592-V08H82 |
| **SARS-CoV-2 Beta Spike** | Sino Biological | 40589-V08B7 |
| **SARS-CoV-2 Beta RBD** | Sino Biological | 40592-V08H59 |
| **SARS-CoV-2 Gamma Spike** | Sino Biological | 40589-V08B10 |
| **SARS-CoV-2 Gamma RBD** | Sino Biological | 40592-V08H86 |
| **SARS-CoV-2 Delta Spike** | Sino Biological | 40589-V08B16 |
| **SARS-CoV-2 Delta RBD** | Sino Biological | 40592-V08H115 |
| **SARS-CoV-2 Omicron BA.1 Spike** | Sino Biological | 40589-V08H26 |
| **SARS-CoV-2 Omicron BA.1 RBD** | Sino Biological | 40592-V08H121 |
| SARS-CoV-2 Omicron BA.5 Spike | Sino Biological | 40592-V08H32 |
| SARS-CoV-2 Omicron BA.5 Spike | Sino Biological | 40592-V08H130 |
| SARS-CoV-2 Omicron BQ.1.1 Spike | Sino Biological | 40589-V08H41 |
| SARS-CoV-2 Omicron BQ.1.1 Spike | Sino Biological | 40592-V08H143 |
| SARS-CoV-2 XBB.1.5 Spike | Sino Biological | 40589-V08H45 |
| SARS-CoV-2 Nucleocapsid | Sino Biological | 40588-V08B |
| HCMV Glycoprotein B | Sino Biological | 10202-V08H1 |
| HCoV-OC43 Spike | Sino Biological | 40607-V08B1 |
| HCoV-NL63 Spike | Sino Biological | 40641-V07E |
| MERS-CoV Spike | Sino Biological | 40069-V08B |
| SARS-CoV-1 Spike | Sino Biological | 40634-V27H |
| A/Brisbane/59/2007 | Sino Biological | 11052-V08H |
| A/California/HKWF609/2007 | Sino Biological | 11699-V08H |
| B/Brisbane/60/2008 | Sino Biological | 40016-V08H |
| A/Singapore/INFIMH-16-0019/2016 | Sino Biological | 40580-V08H |
| A/Brisbane/02/2018 | Sino Biological | 40719-V08H |
| B/Brisbane/60/2008 | Sino Biological | 40203-VNAHC |
| A/Arizona/13/2008 | Sino Biological | 40734-V07H |
| A/Victoria/2570/2019 (H1N1)pdm09-like virus | The Native Antigen Company | REC31949-100 |
| A/Darwin/9/2021 (H3N2)-like virus | The Native Antigen Company | REC32002-100 |
| B/Austria/1359417/2021-like virus (B/Victoria lineage) | The Native Antigen Company | REC32004-100 |
| B/Phuket/3073/2013-like virus (B/Yamagata lineage) | The Native Antigen Company | REC32022-100 |
| A/Wisconsin/588/2019 (H1N1)pdm09-like virus | The Native Antigen Company | REC32000-100 |
| A/Darwin/6/2021 (H3N2)-like virus | The Native Antigen Company | REC32002-100 |
| Human FcγRIIA | Duke Human Vaccine Institute | Custom Order |
| Human FcγRIIB | Duke Human Vaccine Institute | Custom Order |
| Human FcγRIIIA | Duke Human Vaccine Institute | Custom Order |
| Human FcγRIIIB | Duke Human Vaccine Institute | Custom Order |
| Human FcαR | Duke Human Vaccine Institute | Custom Order |
| Streptavidin-PE | Agilent Technologies | PB32-10 |
| Ebola Virus Glyoprotein | IBT Bioservices | 0501-015 |
| Influenza HA | Sino Biological | 11687-V08H |
| LC-LC-Sulfo-NHS Biotin | ThermoFisher | A35358 |
| Pierce EDC No Weight Format | ThermoFisher | A35391 |
| Streptavidin-R-Phycoerythrin | Prozyme | PJ31S |
| **Software and algorithms** | | |
| GraphPad Prism 8 | GraphPad Software, Inc. | Ragon License |
| R Studio V. 4.0.4 | R Project for Statistical Computing | Open Source |
| Flow Jo | BD Bioscience | Ragon License |
| Python V 3.8.8 | MathWorks | Open Source |
| Matplotlib V 3.3.3 | Mathworks with Python | Open Source |
| **Other** | | |
| MagPlex microspheres | Luminex corporation | MC12001-01 |

**Supplementary Table 1. List of reagents and resources used in this study.**
